# Improved procedure for electro-spinning and carbonisation of neat solvent-fractionated softwood Kraft lignin

**DOI:** 10.1038/s41598-021-95352-5

**Published:** 2021-08-10

**Authors:** Inam Khan, Bongkot Hararak, Gerard F. Fernando

**Affiliations:** Sensors and Composites Group, School of Metallurgy and Materials, University of Birmingham, Birmingham, B15 2TT UK

**Keywords:** Biomaterials, Bioinspired materials, Biopolymers

## Abstract

In general, the electro-spinning of lignin requires it to be functionalised and/or blended with synthetic or natural polymers. This paper reports on the use of solvent fractionated lignin-lignin blend to electro-spin BioChoice softwood Kraft lignin. The blend consisted of acetone-soluble and ethanol-soluble lignin in a binary solvent of acetone and DMSO. Solvent fractionation was used to purify lignin where the ash content was reduced in the soluble lignin fractions from 1.24 to ~ 0.1%. The corresponding value after conventional acid-washing in sulphuric acid was 0.34%. A custom-made electro-spinning apparatus was used to produce the nano-fibres. Heat treatment procedures were developed for drying the electro-spun fibres prior to oxidation and carbonisation; this was done to prevent fibre fusion. The lignin fibres were oxidised at 250 °C, carbonised at 1000 °C, 1200 °C and 1500 °C. The cross-section of the fibres was circular and they were observed to be void-free. The longitudinal sections showed that the fibres were not fused. Thus, this procedure demonstrated that solvent fractionated lignin can be electro-spun without using plasticisers or polymer blends using common laboratory solvents and subsequently carbonised to produce carbon fibres with a circular cross-section.

## Introduction

Over the past decade, the use of naturally occurring biomaterials such as lignin and cellulose as an alternative precursor to polyacrylonitrile (PAN) for the production of carbon fibres has been studied extensively^1–5^. PAN continues to be the primary precursor for the production of carbon fibres. However, PAN is derived from petroleum and it is not a sustainable precursor in the long-term^6,7^. There is significant ongoing global interest in identifying and using sustainable, low-cost and environmentally-friendly precursors for the production of carbonised fibres^8–10^. Due to its chemical structure, abundance and high carbon content, lignin has been considered to be a potential low-cost alternative precursor for the production of carbon fibres^7,11–13^. Approximately 50 million tonnes of lignin is produced per year by the paper and pulp industry^8,14^. However, only 1–2% of the lignin produced is used in other industries whilst the rest is burned as fuel for energy generation^14,15^.

Generally, lignin is initially purified by washing it with an acid to reduce the carbohydrate content, inorganic impurities and other contaminants^16–19^. These impurities effect the ability of the lignin/solvent solution to be spun into fibres^16,20,21^. The acid-washed lignin is fractionated, chemically modified or blended with co-polymers to aid fibre spinning and to improve the desired properties^22–25^. The fibres are oxidised and then subjected to prolonged heat-treatment for carbonization.

Natural and the majority of synthetic polymers exhibit a distribution in their molar masses. With reference to aspects of dissolution in solvents, viscous flow and fibre formation, it is desirable to obtain a narrower molar mass distribution. When dealing with polydisperse samples, fractionation is an established method to separate the polymer into a number of fractions where each fraction has a narrow molar mass distribution^26^. The basis for fractionation stems from the Flory–Huggins theory where the interaction parameter χ_1_ can be used to infer the solvating power of the solvent^27,28^. The relationship between the polymer chain length (x_n_) and the critical polymer concentration (χ_1c_) at which phase separation will be observed is given by^27,28^:

1 $${\upchi }_{1c}= {{1 } \mathord{\left/ {\vphantom {{1 } {2 }}} \right. \kern-\nulldelimiterspace} {2 }}+ {{1 } \mathord{\left/ {\vphantom {{1 } {{x}_{n}^{1/2} }}} \right. \kern-\nulldelimiterspace} {{x}_{n}^{1/2} }}+ {{1 } \mathord{\left/ {\vphantom {{1 } {{2x}_{n} }}} \right. \kern-\nulldelimiterspace} {{2x}_{n} }}$$

This leads to the conclusion that if χ_1_ can be adjusted and controlled for a polydisperse polymer solution, appropriate processing conditions can be adjusted to enable a specified molar mass or fraction of the polymer to be precipitated. Fractionation of a polydisperse solution can be achieve in a number of ways but the most common methods involve introducing a non-solvent to the polymer solution or by lowering the temperature. In conventional fractionation, a non-solvent is introduced slowly to the polymer solution that is maintained at a constant temperature. As the concentration of the non-solvent is increased, a critical concentration is reached where the polymer with the longest chains are precipitated; hence, they can be separated using filtration or centrifuge. The concentration of the non-solvent can be increased progressively to achieve the fractionation of the polymer with a narrow molar mass distribution in each fraction^29,30^. Since a linear relationship exists between the interaction parameter and temperature, lowering the temperature of the polymer solution in a controller manner will cause precipitation of the higher molar mass fractions in a manner that is similar to the non-solvent-based approach. The partial solubility of lignin in common organic solvents such as acetone^31^, methanol^32^, ethanol^33^, etc. are also used to facilitate single-solvent fractionation of lignin^34^. Other techniques that have been used to fractionate lignin include, ultrafiltration^35^, segmented continuous flow^36^, combinations involving organic solvents and water^37^, sequential acid fractionation^38^ and membrane separation^39^.

The source, type of lignin and the extraction procedures used influence its processability during fibre spinning^2,40^.

Electro-spinning is a cost-effective method to produce nano-fibre preforms that can subsequently be carbonised^41–44^. The applications of carbonised lignin fibres include nano-composites^45^, tissue scaffolds for biomedical applications^46^, sensors^47^, filtration technologies^21^, lithium ion batteries^48^, sodium ion batteries^49^, fuels cells^50^, double layer capacitors^51^ and dye-sensitized solar cells for energy storage and battery-related applications^52^.

Polymers such as polyethylene oxide, polyvinyl alcohol, polyacrylonitrile and cellulose have been blended with lignin^25,52–57^. One of the reasons for blending lignin with other polymers is to improve its viscoelastic properties which in turn aids electro-spinning^58–60^. The difficulty in electro-spinning lignin is attributed to its molecular weight distribution, cross-linking during processing and intermolecular interaction within the lignin framework. Blending lignin with polymers or plasticisers is thought to facilitate polymer entanglement by disrupting the intermolecular interactions and altering its viscoelastic properties which in turn is said to improve its ability to be electro-spun^58,60–62^. Solvents that have been used frequently for dissolving and electro-spinning lignin fibres include dimethyl formamide and dimethyacetamide^2,24,40^.

This paper reports on the development of a method for electro-spinning a lignin/lignin blend, without using any additives. Lignin was fractionated using acetone and ethanol. The fractionated lignins, with different molecular weights, were dissolved in a 2:1 mixture of acetone and dimethyl sulfoxide prior to electro-spinning. The electros-spun fibres were thermo-oxidised, carbonised and characterised. The carbonised fibres were void-free, unfused and they had a circular cross-section. The fibres were characterised using conventional analytical techniques and compared with data reported in literature.

## Methods

### Characterisation of lignin

The lignins were characterised using traditional techniques including: (i) particle size analysis; (ii) density; (iii) thermo-gravimetric analysis (TGA); (iv) size exclusion chromatography (SEC); (v) differential scanning calorimetry (DSC); (vi) UV–Visible spectroscopy (UV–Vis); (vii) nuclear magnetic resonance spectroscopy (NMR); (viii) viscosity; and (ix) electrical conductivity. The experimental details for each of the above-mentioned methods are presented in the supplementary information section.

Prior to solvent fractionation, the softwood Kraft (BioChoice) lignin was dried in a vacuum oven at 80 °C for 6 h under a reduced pressure of 1 bar to remove moisture and low-molecular weight volatiles. The pre-dried lignin was refluxed with the desired solvent (acetone or ethanol) for 6 h under constant agitation by bubbling argon gas at 30 mL min^−1^. The temperature of the solution was maintained at 56 °C and 75 °C for acetone and ethanol, respectively. The lignin-to-solvent ratio was maintained at 1 g per 15 mL. After refluxing, the solution was cooled to room temperature and filtered under reduced pressure. The solvent from the soluble lignin fraction was evaporated using a rotary evaporator under reduced pressure. The fractionated lignin including the soluble and the insoluble fractions were dried in a vacuum oven at 80 °C for 6 h and stored in an air-tight container until required.

### Ash content of fractionated lignins

The ash content in the pre-dried (moisture-free) lignins was determined using the following procedure. It involved pre-heating alumina crucibles with lids to 525 °C for 60 min and cooling them in a desiccator. Approximately 1 g of lignin was transferred to the pre-dried and pre-weighed crucibles. Alumina lids were placed on the crucibles and they were positioned in a muffle furnace and heated from ambient temperature to 525 °C to carbonise the lignin samples without causing them to ignite. Once the samples had charred, the lid was removed to oxidise lignin samples at 525 °C for 4 h. Upon cooling to room temperature, it was observed that samples had been converted from a black char to white powdery ash. The mass was recorded to the nearest 0.001 g using an analytical balance and the ash content was determined.

### Preparation of lignin solutions for electro-spinning

Solutions of fractionated acetone-soluble (ASL) and ethanol-soluble (ESL) lignin was made using a 2:1 (v/v) ratio of acetone and DMSO. A concentration of 52.8 wt% lignin was identified as an optimum total polymer concentration for electro-spinning. The ASL concentration was kept at 95 wt% with ESL making up the remainder. The solution was homogenised in a nitrogen atmosphere using a magnetic stirrer for 6 h and then stored in an airtight container until required.

### Electro-spinning of lignin solutions

**Figure 1:**
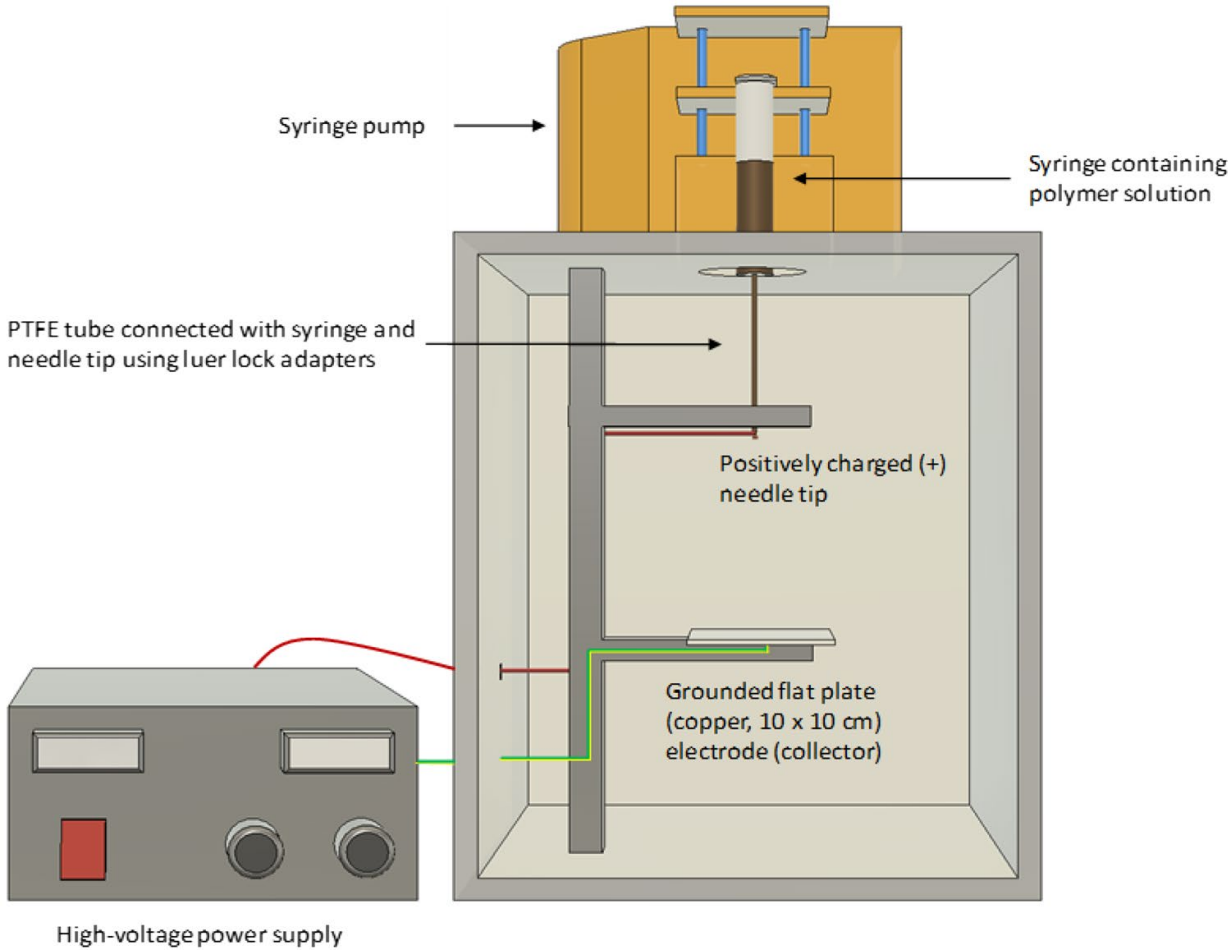
Schematic illustration of electro-spinning setup with a flat plate ground-electrode for collecting randomly oriented lignin fibres. Autodesk (Fusion360), version *2018*. San Rafael, CA, USA. https://www.autodesk.com/.

The electro-spinning of lignin solutions was carried out using a custom-built electro-spinner consisting of a disposable syringe and needle assembly (Teflon tube with luer lock adapter, Cole Parmer), a controllable feed liquid dispenser (AL1010, World Precision Instruments) and a flat-tip needle of 25 G (0.254 mm diameter, Adhesive Dispensing). A schematic illustration of the electro-spinning unit that was used to produce lignin fibres is shown in Fig. 1. The needle was connected to the positive terminal of a high-voltage power supply (Laboratory bench power supply, Genvolt). The electro-spun fibres were collected on an aluminium foil placed on top of a grounded copper plate (10 × 10 × 0.5 cm). The distance from the tip of the needle to the collector plate was kept at 12 cm and the applied voltage was 12 kV. The polymer solution was dispensed at 0.1 µl min^-1^. The temperature of the chamber was maintained between 25 and 30 °C with a relative humidity of 30–35%. The electro-spinning operation was carried for 3 min.

### Thermo-stabilisation and carbonisation of electro-spun lignin fibres

Prior to thermo-stabilisation, the electro-spun ASL-ESL lignin fibres were dried in vacuum oven at 140 °C for 6 h to remove excess solvent from the electro-spun fibres. The pre-dried lignin fibres were transferred into a tube furnace (Pyrotherm) in a graphite crucible and heated to 100 °C and held for 1 h. The fibres were then heated to 150 °C, held for 1 h followed by heating to 250 °C where they were held for another 1 h. During the thermo-stabilisation step, a heating rate of 0.5 K min^-1^ was used under an air flow of 50 mL min^−1^.

The thermo-stabilised ASL-ESL lignin fibres were carbonised at 1000 °C, 1200 °C and 1500 °C. These samples were heated at 5 K min^−1^ under a nitrogen gas flow rate of 50 mL min^−1^. The fibres were held isothermally at each temperature for one hour before cooling to room temperature.

### Fibre morphology

A TM3030 PLUS (Hitachi, Japan) SEM was used to characterise the surface and cross-section morphology of the lignin samples that were carbonised at 1000 °C, 1200 °C and 1500 °C. The SEM was operated with an acceleration voltage of 15 kV. The sample was mounted on SEM stub using an adhesive carbon tape and coated with Au/Pd for 3 min using a current of 25 mA and a vacuum of 1 mTorr.

### Fibre diameter distribution

The fibre diameter distribution of the lignin fibres, before and after carbonisation, was acquired using ImageJ analysis software. Three representative micrographs at a magnification of 2500 were selected for each sample and the diameter distribution was determined using one hundred individual measurements for each image.

### Electrical conductivity

The electrical properties of the carbonised electro-spun lignin fibres were measured using a 4-point probe of RM3000 (Jandel Engineering Limited). Prior to measuring the electrical resistivity, the equipment was calibrated using a Jandel resistivity standard (Serial no. 74452, Jandel Engineering Limited). The carbonised lignin fibre with a width of 0.5 cm were mounted on a clean glass slide. The sheet or surface resistance of fibres that were carbonised at 1000 °C, 1200 °C and 1500 °C was measured. The sample thickness of electro-spun carbonised mat was averaged from five individual measurements. The spacing or distance between the probes was set at 0.1 cm and the sample measurements were carried out at 24–25 °C and with a relative humidity of 38–40%. Five measurements of the surface resistance (R) were made at different locations on the samples. The resistivity (Ω-cm) using four-point probe method can be measured according to the Eq. (2)^63,64^:

2 $$\uprho = \frac{{R{ }}}{L}A$$

where *ρ* is the resistivity and *L* is the distance between the probes (0.1 cm) and *R* is the electrical resistance; *A* is the cross-sectional area of the sample.

### Raman spectroscopy

An inVia confocal Raman Microscopy (Renishaw, UK) equipped with a 488 nm laser diode was used to observe the graphitic structure of the carbonised electro-spun fibres. The samples were mounted on the glass microslide. Raman spectra were acquired over a spectral range of 320–3200 cm^−1^ using 100 scans per sample at 10% laser power. The band intensities including the peak area (A) and the peak height (I) were determined. The ratio of intensities of D to G are represented by I_D_/I_G_ whilst A_D_/A_G_ shows the ratio of areas^65,66^.

## Results and discussion

Particle size distribution for the as-received lignin was in the range 0.03–158 µm with d(10), d(50) and d(90) percentiles corresponding to 1.38, 8.75 and 52.47 µm respectively. The density was measured to be 1380 kg/m^3^. With reference to the TGA data shown in Figure S1 (see supplementary information), the lignin char obtained after thermal treatment to 900 °C in argon was used as a screening method to infer the potential for the materials to be used as precursors to produce carbonised electro-spun fibres. The char content from the TGA data at 900 °C for the as-received (ARL), acetone-soluble (ASL), acetone insoluble (ALR), ethanol-soluble (ESL) and ethanol-insoluble lignin (ELR) were 43.4, 35.9, 37.4, 33.9 and 38.6% respectively. The derivative of the mass-loss versus temperature (DTG) traces for the ARL, ASL and ESL lignin show minor peaks just below 100 °C, presumably due to the loss of low-molecular components in the lignin, evaporation of residual solvent and absorbed moisture. Peaks of slightly larger magnitude are observed with maxima at ~ 150 °C for the ASL and ESL whereas that for the ALR and ELR peaked at ~ 200 °C; the ARL did not show a similar magnitude in the peak heights. The observed increase in the rate of degradation above 200 °C for the ASL and ESL, and above 250 °C for the ALR and ELR has been attributed to the degradation of hemi-cellulose^67^ or lignin-carbohydrate complexes^17^. The rapid increase in the mass-loss after 350 °C is due to the degradation of lignin^68^.

The number and weight average molar masses for the lignins investigated in this study are summarised in Table S1 (supplementary information).

The SEC traces for the soluble fractions (ASL and ESL) including as-receive lignin (ARL) are presented in Fig. S2(a) and those for the insoluble fractions (ALR and ELR) including as-receive lignin (ARL) are shown in Fig. S2(b); the data for the ARL have been duplicated in both the figures to enable comparison. The PDI is an indication of the heterogeneity of polymeric materials and the aspiration is that fractionation will yield relatively homogeneous fraction with a lower PDI when compared to the parent polymer. Table S1 shows that the PDI for the ASL and ESL are lower than that for the insoluble fractions and the ARL. The data obtained here shows a similar trend to that reported by Karaaslan et al*.,* for BioChoice lignin^31^.

DSC analyses were carried out to study the evolution of the T_g_ as a function of three successive scans starting with the ARL. Typical DSC traces for the ARL, ASL, ALR, ESL and ELR are shown in Fig. S3. The first DSC scan for all the lignins show the presence of a large endothermic peak between 20 and 120 °C. This is attributed to the evaporation of absorbed water and low molecular weight components in the lignin including residual solvent. The T_g_ from the second heating DSC scan for the lignins are presented in Table S1 where the correlation between the solvent used, ash content and the T_g_ is apparent. A summary of the T_gs_ is presented in Fig. S4 where it is clear that the T_g_ increases from the first to the third scan. This shift in the T_g_ after each scan could be due to the sequential heating of lignin to 250 °C (T > T_g_) causing a structural change and thus resulting in a higher T_g_ in the subsequent scan. This may be due to the reduction in the free-volume brought about inter and intra-molecular reactions including cross-linking^69–75^. T_g_ data were used to determine the drying and oxidation temperature regimes for subsequent experiments involving the electro-spun fibres; this was necessary to prevent the fibres from fusing.

With reference to UV–Vis spectroscopy, typical spectra for the lignins dissolved in DMSO is shown in Figure S5, the structural moieties of lignin give different absorption maxima and extinction coefficients. The conjugated aromatic (non-condensed phenolic units) groups exhibit π-π* electronic transitions at a given wavelength. For the G-lignin (guaiacyl) unit this transition occurs at ~ 280 nm and its extinction coefficient is found to be three times higher than that of the S-lignin (syringyl) unit. In the case of the S-lignin (syringyl) unit, this transition is seen at a lower wavelength due to the additional substitution of the methoxyl (OMe) groups at the C-5 position; this shifts the absorption maxima to between 270 and 273 nm^17,18,76–79^. Since softwood Kraft lignin is mainly comprised of G-lignin units, the absorption maxima are observed at approximately 280 nm.

The absorption maximum at ~ 320 nm is ascribable to the π–π* electronic transition assigned to the conjugated carbon (C=C) bonds^18,80^. Another absorption maximum for the lignin samples is observed at 340 nm. This absorption corresponds to α-carbonyl groups and esters of hydroxycinnamic acids (e.g., ferulic acid). The calculated maximum absorption coefficients at given wavelength are shown in Table S2. The higher extinction coefficients are indicative of a higher lignin content^78^. All the soluble fractions specifically the acetone soluble lignin fraction (ASL) exhibit higher extinction coefficient in comparison to the as-received lignin (ARL). The extinction coefficients for the insoluble lignin fractions are relatively similar or slightly lower as compared to the parent lignin. The higher absorbance and extinction values for the soluble fraction could be related to the lower concentration of impurities^18,80,81^; this assumption correlates well with the reduced ash content upon fractionation as shown in Table S1. This provides further evidence to support the view that the solvent fractionation can be used to refine and purify lignin before undertaking secondary operations such as fibre spinning.

In recent years, heteronuclear single quantum coherence spectroscopy has been used extensively to characterise lignin. In the current study, the ^31^P NMR spectra (see Fig. S6) data summarised in Table S3 show that the soluble lignin fractions have a low concentration of aliphatic hydroxyl groups in comparison with the insoluble lignin fractions seen in Table S3. The reduction in the aliphatic hydroxyl groups and the increase in the phenolic hydroxyl groups correlates with data presented in the literature^26,82–84^.

The soluble lignin fractions have a noticeably higher composition of phenolic units, particularly of the predominant softwood G-lignin (guaiacyl) moieties, when compared to the insoluble lignin fractions. The carboxylic acids content is also found to be higher in the soluble fractions compared to the insoluble lignin fractions. This observation agrees with the data reported in the literature for softwood Kraft lignin^26,82,83^.

The soluble fractionations of lignin with acetone and ethanol yielded 56% and 38% respectively. As seen in Table S1 (supplementary information), the ash concentration in the soluble fractions were in the range 0.1–0.11% whereas that for the as-received lignin was 1.24%. The ash content for the insoluble lignin fraction was 1.99–2.17%. The conventional approach to remove the inorganic content in lignin is to treat it with acids. This was carried out using sulphuric acid on the as-received lignin and the ash content was found to be 0.34%. The removal of the inorganic content is deemed necessary to reduce the carbohydrates content, inorganic impurities and volatile contaminants^16–19^. These impurities affect the fibre spinnability and influence the physico-chemical properties^16,20,21^.

With reference to the seminal works by Crestini et al*.,* the conclusions that they reached was that acetone-soluble softwood Kraft lignin is more branched and of a lower molecular weight when compared to the acetone insoluble fraction^85^. This collaborates with the findings in this study with regard to the molecular weight distribution, the PDI and the glass transition temperature. This then leads to the observation presented in Table S4 (supplementary information) where some combination of processing parameters enabled the production of electro-spun fibres whereas others did not. This is not an easy question to answer with any certainty as the majority of the electro-spinning processing parameters are interrelated and some of them were selected for practical reasons. For example, a binary solution of acetone and DMSO was selected primarily to address the rapid evaporation of acetone but it also served to create a “skin” on the electro-spun fibres. DMSO has a boiling point of 189 °C compared to 56 °C for acetone. This meant that: (a) the stretching of the fibres during electro-spinning due to the whipping action of the polymer jet had to enable the acetone to evaporate rapidly thus enabling a “skin to be formed on the fibre before the DMSO-rich filament is deposited on the grounded plate; and (b) a higher drying temperature had to be used to remove the DMSO before oxidation of the lignin. A detailed set of experiments were designed and executed to identify the parameters that enabled the DMSO in the fibres to be driven off without the structure of the fibre collapsing, leading to fused fibres or ribbons. The details of the drying regime will be reported in a subsequent publication but in summary it was found that the heat treatment regime adopted in this paper prevented fibre fusion during oxidation of the lignin prior to carbonisation. The selection of the solvents was a simple choice as the remit was to use common low-cost laboratory solvents and/or those that are not toxic. The ratio for the solvents was determined on a trial-and-error basis. Different solvents ratios of acetone/DMSO such as 1:1 and 3:1 including neat acetone were evaluated in addition to the experiments listed in Table S4 (supplementary information). Neat acetone and the 1:1 ratio of acetone/DMSO resulted in the clogging of the needle whilst the 3:1 ratio resulted in the formation of beaded and non-continuous fibres. The 2:1 acetone/DMSO in conjunction with experiment numbers 3, 5 and 6 stated in Table S4 enabled continuous electro-spinning for 3 min after which the experiments were terminated. The assessment criteria were (i) continuous fibres without beads, (ii) unfused fibres, (iii) fibres with a circular cross-section, (iv) defect-free fibres (transverse section) and (v) smooth fibre surface. With respect to finding an appropriate fractionated lignin ratio for acetone and ethanol, three different ratios of acetone and ethanol (Table S4) were assessed. The rationale behind using a higher ASL lignin fraction with ESL were: (i) that the former has a marginally higher char content upon pyrolysis as seen in the TGA data shown Figure S1; (ii) the latter has a lower molecular weight distribution and PDI (Table S1) and lower T_g_ (Figure S3 and S4). It was assumed that the ESL will be chemically and thermodynamically compatible with the ASL and that it would “plasticise” the ASL without needing the inclusion of a polymer blend. Whilst unfractionated (hardwood) lignin has been electro-spun using a co-axial electro-spinning setup^86,87^, at the time of writing, the authors were not aware of any previous publications where a conventional electro-spinning experimental setup was used to electro-spin lignin/lignin blends.

Scanning electron micrographs representing the electro-spinning experiments cited in Table S4 are shown in Fig. S7 and S8 (supplementary information). With reference to Figure S7, (a-b) show solvent-rich fibre morphology for experiment-1 where the polymer concentration was the lowest; (c-d) show the presence of beaded fibres and fibres with a range of diameter and these were obtained from experiment-2; (e–f) show fibres with a smooth surface that are circular in appearance and these correspond to experiment-3 where the solution viscosity was 0.42 Pa.s; (g-h) show the occurrence of fused fibres that were produced in experiment-4. In Fig. S8, (a-b) and (c-d) show fibres with a blemish-free surface along with a circular morphology and these correspond to experiment-5 with a 90% ASL/10% ESL and in experiment-6 where the ASL:ESL ratio was 70:30), respectively.

### Electro-spinning of ASL/ESL

**Figure 2:**
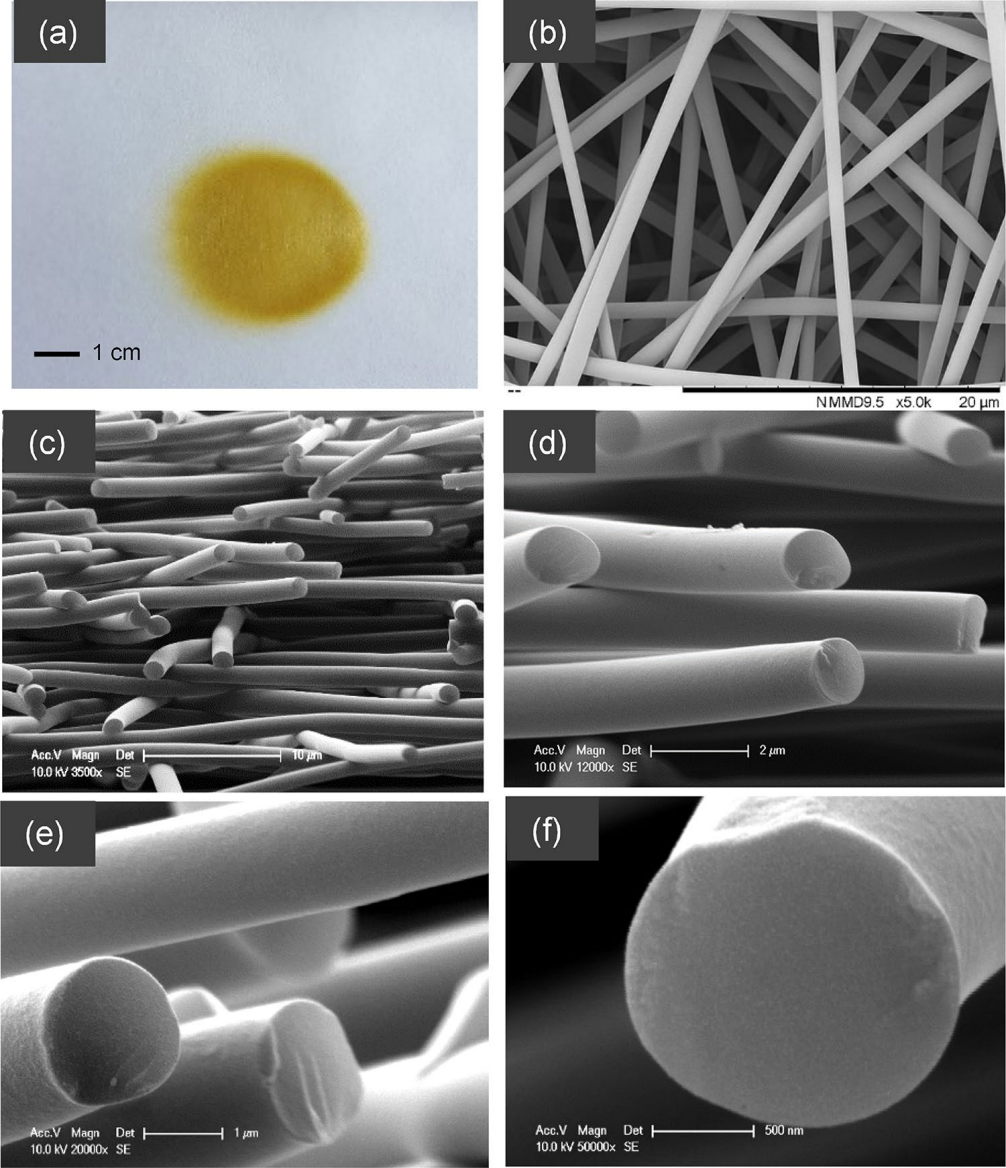
(**a**–**f**) Electro-spun lignin fibres using 95%ASL–5%ESL in acetone/DMSO: (**a**) macroscopic appearance of the deposition area (randomly oriented fibres); and (**b**–**f**) magnified SEM micrograph of fibres produced using the 52.8 wt% total polymer solution concentration.

The fibres were spun on to a flat plate collector and a schematic illustration of electro-spinning setup was shown in the experimental method section. A macroscopic image of the deposition area of the electro-spun lignin is shown in Fig. 2a–f. Pale brownish fibres were deposited over a diameter of 3–4 cm. The fibres within this area were orientated randomly. Figure 2b–f show representative micrographs for the 52.8% total polymer concentration (with 2:1 ratio of acetone/DMSO) where the fibres are unfused and orientated randomly. Figure 2e,f shows high-magnification SEM micrographs for the transverse section where it can be seen the fibres are void-free and with a smooth surface.

**Figure 3:**
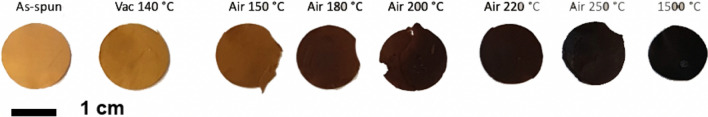
Colour changes in the electro-spun ASL-ESL lignin fibres before and after heat treatment at specified temperatures.

Electro-spinning of fractionated lignin without any additives was demonstrated successfully for the first time (Fig. 2). This is significant as softwood Kraft lignin was found to be unsuitable for the production of carbon fibres and for use in biorefineries due to its higher content of impurities^18,19^. The electro-spun ASL-ESL lignin fibres were dried in a vacuum oven at 140 °C and then subsequently thermo-stabilised in air at 250 °C. The thermo-stabilised fibres maintained their form and structural shape. The change in structural and physical properties during thermo-stabilisation will be discussed in subsequent publication. The thermo-stabilised lignin fibres were carbonised in a tube furnace at 1000 °C, 1200 °C and 1500 °C. The colour change observed before and after the specified heat-treatment of the electro-spun lignin fibres is shown in Fig. 3. The as-spun fibres turn from pale yellow/brown to dark brown and eventually to black upon carbonisation.

**Figure 4:**
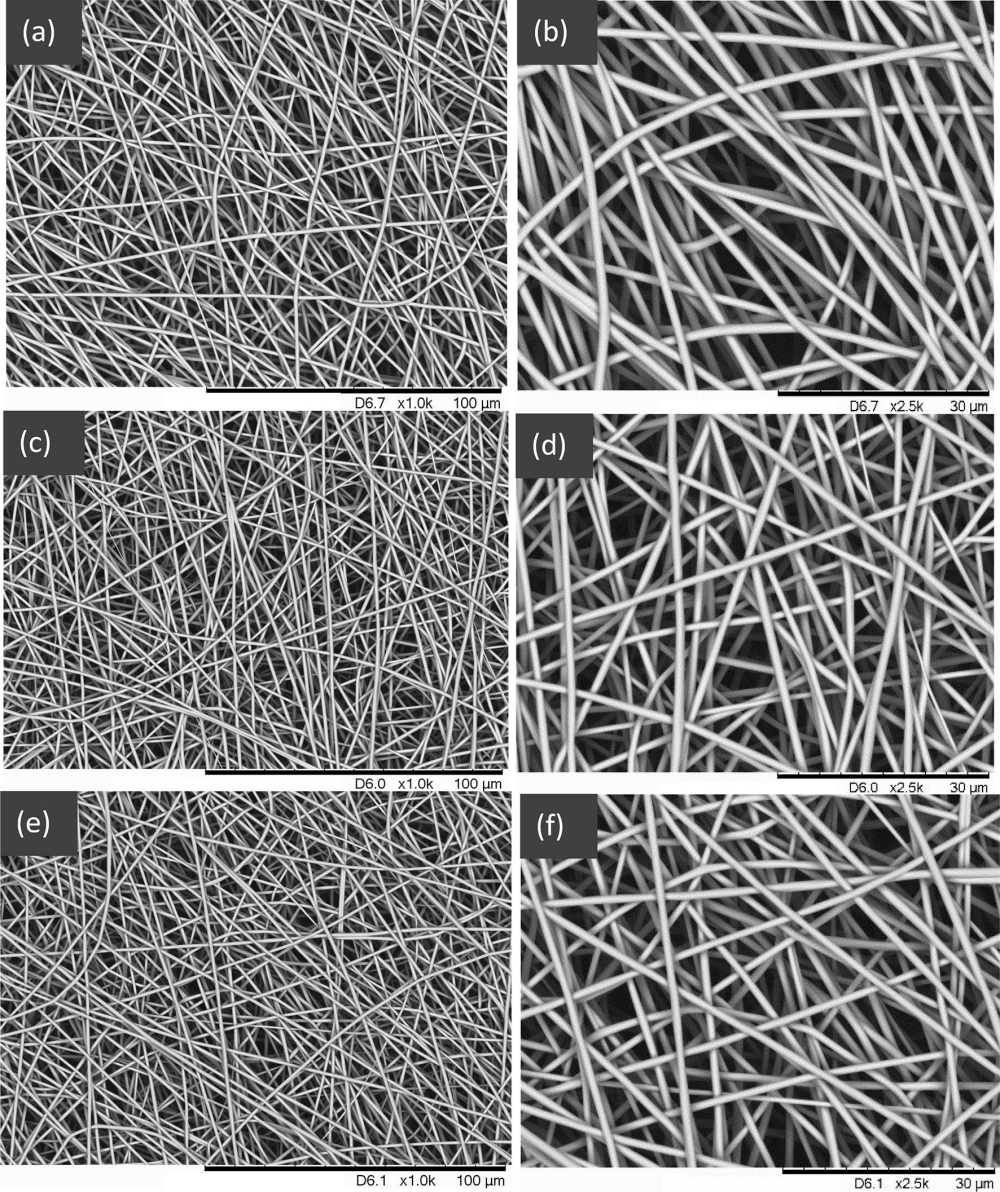
Electro-spun and carbonised (ASL-ESL) lignin fibres after carbonisation at 1000 °C (**a**,**b**), 1200 °C (**c**,**d**) and 1500 °C (**e**,**f**) with magnifications of × 1000 and × 2500.

SEM micrographs of the carbonised lignin fibres (ASL-ESL) at 1000 °C, 1200 °C and 1500 °C are shown in Fig. 4a–f. The surface morphology of the fibres is seen to be smooth, circular in cross-section and unfused. This demonstrates conclusively that electro-spun lignin fibres can be obtained without the use of any processing aids or synthetic polymer blends. In other words, this represents the production of electro-spun fibres using 100% lignin.

**Figure 5:**
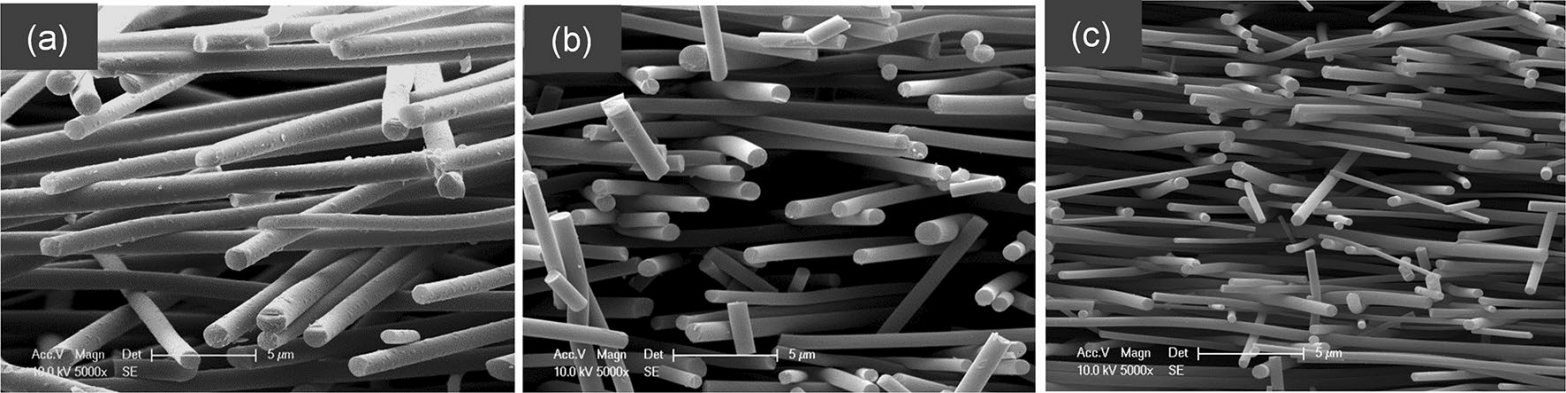
(**a**–**c**) Micrographs showing transverse sections of electro-spun (ASL-ESL) lignin fibres after carbonisation at 1000 °C, 1200 °C and 1500 °C respectively.

**Figure 6:**
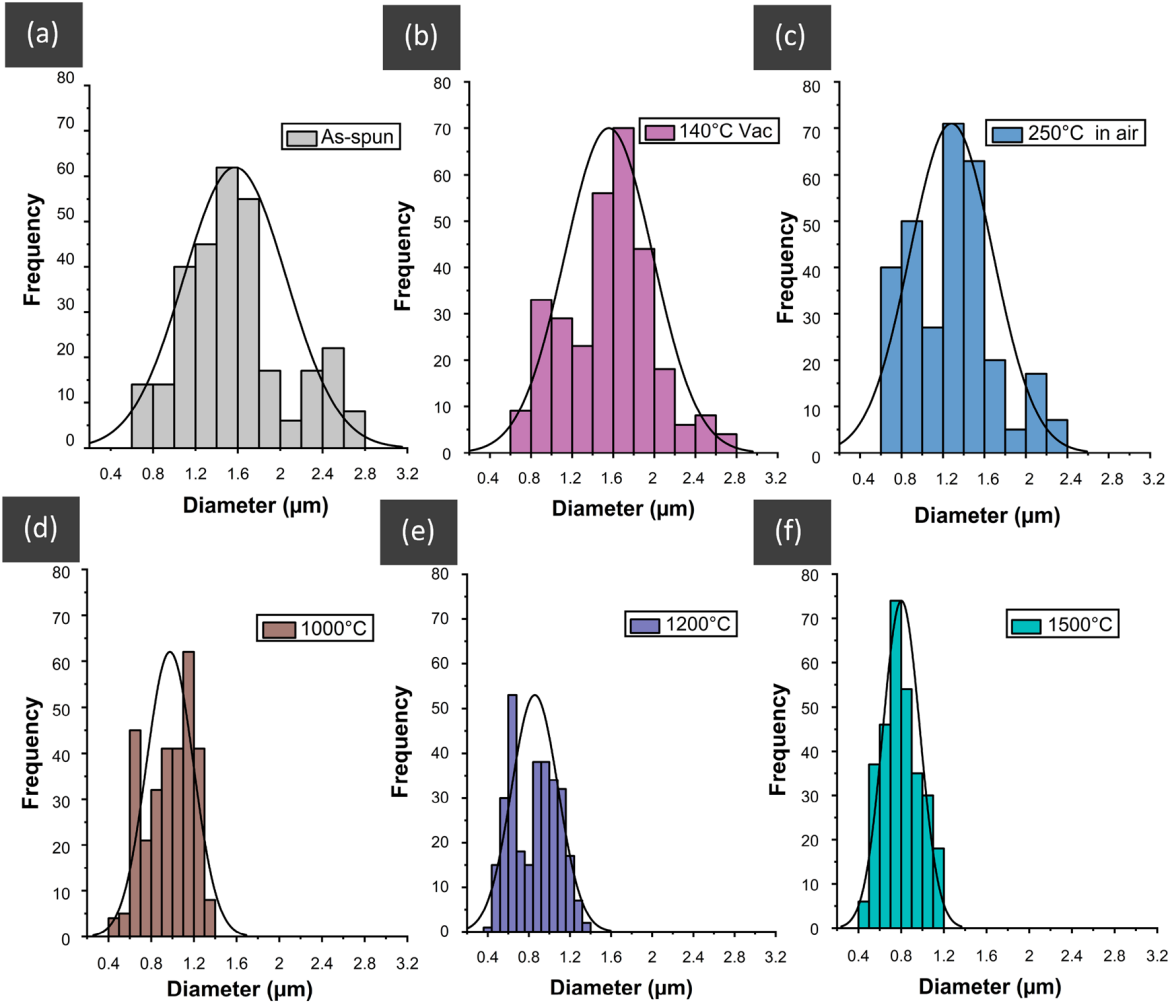
Histogram plots for the diameter distribution for the electro-spun (ASL-ESL) lignin fibres: as-spun (**a**), vacuum-heated at 140 °C (**b**), thermo-oxidative stabilised at 250 °C (**c**), and carbonised at 1000 °C (**d**), 1200 °C (**e**) and 1500 °C in nitrogen (**f**). The histograms have been overlaid with a normal diameter distribution curve for each data set. Origin (Pro), version *2020*. OriginLab Corporation, Northampton, MA, USA. https://www.originlab.com/.

Transverse section of the electro-spun (ASL-ESL) lignin fibres that were carbonised at 1000 °C, 1200 °C and 1500 °C is shown in Fig. 5. These micrographs demonstrate that the fibres are not fused and that their cross-section is circular. Figures 5a–c and 6a–f show that the fibre diameter decreases as a function of the carbonisation temperature. This is expected due to shrinkage and mass-loss during carbonisation. Figure 5a–c show the presence of fractured fibres. This was possibly caused when the electro-spun preform was fractured in liquid nitrogen to obtain transverse sections. However, fracture caused by fibre shrinkage during carbonised cannot be ruled out.

### Fibre diameter distribution

The fibre diameter distribution for the electro-spun ASL-ESL lignin fibres in the as-spun and after specified heat treatments are shown in Fig. 6a–f. The data sets are represented as histogram plots with an overlaid normal distribution curve. The diameters for the electro-spun samples represented in Fig. 6 are as follows: (a) as-spun 0.6–2.8 µm; (b) vacuum-dried 0.6–2.8 µm; (c) thermo-stabilised lignin fibre in air at 250 °C: 0.6–2.4 µm; (d) carbonised lignin fibres in nitrogen at 1000 °C 0.6–1.4 µm; (e) 1200 °C: 0.6–1.4 µm and (f) 1500 °C: 0.6–1.2 µm. The as-spun and vacuum dried lignin fibres showed the widest distributions ranging between 0.6–2.8 µm. There is a noticeable change in diameter for the air thermo-stabilised lignin fibre diameter at 250 °C. The most significant change in the diameter distribution for the electro-spun fibres was observed during the carbonisation stage. The narrowest diameter distribution (0.4–1.2 µm) was obtained for the lignin fibres that were carbonised at 1500 °C.

### Electrical conductivity of electro-spun ASL-ESL carbonised lignin fibres

The electrical properties of the carbonised lignin samples are shown in Table 1. The electrical properties of the solid carbonised lignin fibres are important for determining their suitability for energy storage applications such as electrodes for dye-sensitised solar cells, batteries, fuel cells, capacitors and super capacitors. As seen in Table 1, the electrical conductivity of the lignin fibres increased with carbonisation temperature from 1000 °C to 1200 °C and 1500 °C. The lignin fibres that were carbonised at 1500 °C show electrical conductivity comparable to those reported in literature for softwood Kraft lignin (230 S m^−1^)^52,66^. This suggests that the carbon fibre produced from 100% lignin, without any binder as in the current case, are of comparable quality to those reported in literature.

**Table 1:** Electrical properties of the carbonised ASL-ESL lignin fibres that were carbonised in nitrogen at 1000 °C, 1200 °C and 1500 °C for 1 h.

Samples	Resistivity (Ω m)	Electrical conductivity (S m^−1^)
1000 °C	0.96 $\pm$ 0.14	105.64 $\pm$ 14.86
1200 °C	0.51 $\pm$ 0.05	197.18 $\pm$ 20.40
1500 °C	0.49 $\pm$ 0.06	205.80 $\pm$ 24.33

### Raman spectroscopy of electro-spun ASL-ESL carbonised lignin fibres

**Figure 7:**
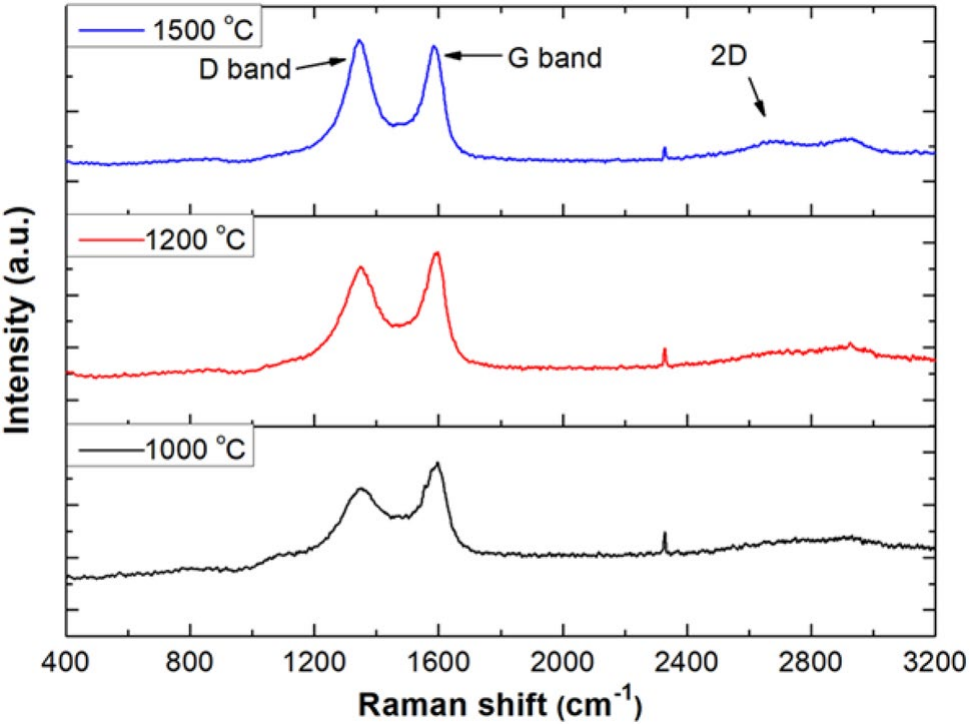
Raman spectra of electro-spun and randomly orientated lignin fibre mats that were carbonised at 1000 °C, 1200 °C and 1500 °C in nitrogen for 1 h. Origin (Pro), version *2020*. OriginLab Corporation, Northampton, MA, USA. https://www.originlab.com/.

Raman spectroscopy was used to evaluate the structural changes in the lignin samples as a result of carbonisation. Raman spectra of the lignin fibres that were carbonised at 1000 °C, 1200 °C and 1500 °C are shown in Fig. 7. There are two distinct peaks present in the Raman spectra for the carbonised lignin fibres. These characteristic peaks correspond to D and G-bands which are typical of lignin and PAN-based carbon fibres. The D-band which appears at ~ 1350 cm^−1^ is attributed to the breathing modes of carbon atoms in aromatic rings. The G-band which appears at ~ 1600 cm^−1^ is ascribed to the in plane stretching of *sp*^*2*^ carbon hybridized bonds (C=C) in the aromatic rings^2,65^. Therefore, in general, the D-band indicates disorder or defects in the graphitic structure and the G-band represents *sp*^*2*^ ordered graphitic carbon.

The small peak between 1000 and 1200 cm^−1^ could be attributed to *sp*^*3*^ hybridised carbon^88^. The emergence of significant new peaks at approximately 2700 and 2900 cm^−1^ is observed as the carbonisation temperature was increased from 1000 to 1500 °C. This band is a result of second order resonance from the D-band and it is normally referred to as 2D band. This 2D band is a characteristic feature of increased stacking in the layered graphitic sheets^4,89^.

The relative positions of the D and G-bands in the Raman spectra with an analysis of the ratios of two peaks is shown in Table 2. The intensity of the D and G-bands increases with the processing temperature. The intensity (I_D_/I_G_) and area (A_D_/A_G_) ratios of the two bands increase with temperature. This suggests that more disorder is introduced in the graphitic structure with increasing carbonisation temperature from 1000 to 1500 °C. This is said to indicate the existence of turbostratic graphite which is thought to be composed of highly condensed aromatic structure between the amorphous carbon and graphite^65,90^. The increase in the ratio of I_D_/I_G_ is directly in contrast to carbon fibres made from PAN where this ratio is seen to decline with the increasing carbonisation temperature^91–93^. However, these findings are in complete agreement with reported Raman results on the carbon fibres obtained from lignin^4,20^. Moreover, it is reported that the higher molecular weight of lignin enhances the graphitic structure and mechanical performance^94^.

**Table 2:** Analysis of the position of the D and G-band in the Raman spectra for the carbonised lignin fibres and the full width at high maximum (FWHM) for these bands along with their intensity and area ratios.

Sample	D-band (cm^-1^)	G-band (cm^−1^)	Width (FWHM) (cm^−1^)		I_D_/I_G_	A_D_/A_G_
			D-band	G-band		
1000 °C	1352.91	1597.02	164.52	135.18	0.82	0.96
1200 °C	1348.61	1597.02	146.19	89.85	0.91	1.07
1500 °C	1342.88	1583.11	107.59	77.97	1.04	1.13

The FWHM of the D and G- bands is attributed to the degree of structural disorder. It is seen in Table 2 that the D and G-bands experience a decrease in the FWHM values, but G-band shows more narrower line width as the carbonisation temperature is increased. This indicates the lignin sample carbonised at higher temperature start to attain a crystallite graphitic structure^4,95^.

## Conclusions

The solvent fractionation with acetone and ethanol lead to the reduction in ash content (1.24 to 0.1%), average molecular weight (7400 to 5400 g/mol) and glass transition temperature (155 to 134 °C) as determined using molecular weight distribution curves and differential scanning calorimetry (shown in supplementary Fig. S2–S4 and Table S1). The observed trend in the glass transition temperature could be attributed to the lignin fractions having different molecular weight distributions and this agrees with that reported in the literature. The soluble fractions tend to have higher phenolic-to-aliphatic lignin moieties where the concentrations of G-lignin units were prominent. The electrospinning of 100% lignin without any additives was demonstrated successfully for the first time by using non-toxic green solvents (acetone/DMSO). The procedures reported in this study will enable the sustainable production of carbon fibres using lignin and green solvents. The electro-spun and carbonised lignin fibres were void-free with a circular cross-section. The electrical conductivity was comparable to those reported in the literature. An increase in the carbonisation temperature lead to an increase in the graphitic structure as indicated by a narrower FWHM for the G-band in the Raman spectra. The diameter of the lignin fibre was found to reduce with the increase in carbonisation temperature. The mean fibre diameter observed after carbonisation at 1500 °C was 0.8 µm ± 0.4.

## Supplementary Information

Supplementary Information.
