# Supplementary material for: Improved procedure for electro-spinning and carbonisation of neat solvent-fractionated softwood Kraft lignin
Source: Sci Rep. 2021 Aug 10;11:16237. doi: 10.1038/s41598-021-95352-5 (PMC8355243; doi:10.1038/s41598-021-95352-5)
Supplement: Supplementary file 1 — Supplementary Information. [file 41598_2021_95352_MOESM1_ESM.docx]

Improved procedure for electro-spinning and carbonisation of neat solvent-fractionated softwood Kraft lignin

Inam Khan, Bongkot Hararak and Gerard F. Fernando*

Sensors and Composites Group, School of Metallurgy and Materials, University of Birmingham, Birmingham, B15 2TT, UK.

**Supplementary Information**

**1.0 Experimental methods:** Characterising the lignins used in the current study

**1.1 Particle size analysis:** A Malvern Mastersizer 2000 (Malvern Panalytical, UK) laser particle size analyser was used to determine the particle size distribution of the lignin samples. Five measurements were obtained for each sample. The experiments were carried out at 25°C.

**1.2 Density:** A gas pycnometer (AccuPyc II 1340, Micromeritics, USA) was used to measure the density of the as-received softwood Kraft lignin. Five measurements were made for each sample.

**1.3 TGA:** Thermogravimetric analysis (TGA) experiments were performed on a Netzsch STA 449 (Netzsch, UK). Approximately 10 mg of the sample was placed in a platinum pan and transferred into the sample chamber. The sample was heated from 25 to 900°C at 10 K/minute under an argon atmosphere. The gas flow rate was maintained at 50 cm^3^/minute throughout the experiment.

**1.4 DSC:** The differential scanning calorimetry (DSC) experiments were performed on a power compensated Diamond DSC (Perkin Elmer, UK) was used to determine the glass transition temperature (T_g_) of the as-received, soluble and insoluble lignin fractions. These experiments were conducted in a nitrogen atmosphere where the flow rate was maintained at 50 cm^3^/minute. The samples were heated from 25-250°C at 10 K/minute. The samples were cooled to 10°C at 10 K/minute and two successive heating/cooling cycles were performed.

**1.5 UV-Vis spectroscopy:** A Camspec M550 Double Beam Scanning Spectrophotometer was used to acquire ultraviolet spectra of lignin solutions. Approximately 0.5 mg of lignin was dissolved in 20 ml of DMSO at 20°C. Approximately 3 ml of the liquid was transferred to a quartz cuvette (6030-UV, Hellma UK LTD) with a path length of 1 cm. A reference spectrum was obtained using neat DMSO prior to evaluating the sample. The scanning range was from 190-500 nm with a scan interval of 0.5 nm.

**1.6 ^31^P NMR:** Quantitative phosphorous nuclear magnetic resonance (^31^P NMR) spectroscopy of as-received and lignin fractions was undertaken using established method^4-8^. ^31^P NMR spectra were obtained using a Bruker NEO console with an operating frequency of 500 MHz and equipped with nitrogen-cooled cryoprobe. A total of 256 scans were acquired at 25 °C with relaxation delay of 25 seconds. Approximately 40 mg of the pre-dried lignin was added to specimen vial followed by a 1 mL of mixture of anhydrous pyridine and deuterated chloroform (1.6:1, v/v respectively) at room temperature. 50 µL of a relaxation agent, chromium (III) acetylacetonate was added followed by the addition of 100 µL of the internal standard, N-hydroxy-5-norbornene-2,3-dicarboximide. 100 µL of the phosphorylating reagent, 2-chloro-4,4,5,5-tetramethyl-1,3,2- dioxaphospholane (TMDP) was added to the mixture and stirred manually and transferred to an NMR tube.

**1.7 Gel permeation chromatography:** The samples, including the standards, were dissolved in dimethylformamide (DMF) containing 5 mmol of NH_4_BF_4_. The instrument was calibrated using polymethylmethacrylate (PMMA) standards with molecular weights in the range of 550-955,000. The molecular weight distribution was obtained using GPC (Agilent 1260 Infinity II-MDS). This instrument was equipped with refractive index detector and UV wavelength of 280 nm). DMF was used as the mobile phase eluent with a flow rate of 1 ml/minute at 50°C. A 100 µL of dissolved lignin sample, in DMF, with a concentration of 1 mg/mL was injected into the column.

**1.8 Viscosity:** A HR-1 discovery hybrid rheometer (TA instruments, USA) was used to determine the viscosity of the lignin solutions using a 40 mm diameter parallel-plate geometry. The viscosity was determined by subjecting the polymer solution to a shear rate in the range of 0.1 to 100 s^-1^ at 25°C. A solvent trap was used to minimise evaporation of the solvents.

**1.9 Electrical conductivity:** A Jenway 4510 (Cole-Parmer, UK) conductivity meter was used to measure the conductivity of the polymer solutions. The conductivity meter was calibrated at 25°C using a standard solution of sodium chloride supplied by Hannah instruments (USA). Ten measurements of lignin solutions were obtained and averaged.

**2.0 Supplementary Results**

TGA and DTG

**Supplementary Figure S1** TGA and DTG traces for the lignins: (a) soluble; and (b) insoluble fractions. The data for the ARL have be reproduced in both figures to enable comparison. Origin (Pro), version 2020. OriginLab Corporation, Northampton, MA, USA. https://www.originlab.com/

**SEC**

**Supplementary Figure S2** Molecular weight distribution traces for the lignins: (a) soluble; and (b) insoluble lignin fractions. The data for the ARL have been plotted in both the figures for ease of comparison. Origin (Pro), version 2020. OriginLab Corporation, Northampton, MA, USA. https://www.originlab.com/

| **Samples** | **M_w_**  **(g/mol)** | **M_n_**  **(g/mol)** | **PDI**  **(Mw/Mn)** | **T_g_**  **(°C)** | **Ash**  **%** |
| --- | --- | --- | --- | --- | --- |
| As-received lignin (ARL) | 7400 | 3400 | 2.18 | 154.7 | 1.26 |
| Acetone soluble lignin (ASL) | 6700 | 3400 | 1.98 | 146.7 | 0.10 |
| Acetone insoluble lignin residue (ALR) | 17700 | 7500 | 2.36 | 181.2 | 2.17 |
| Ethanol soluble lignin (ESL) | 5400 | 3100 | 1.74 | 134.2 | 0.11 |
| Ethanol insoluble lignin residue (ELR) | 16200 | 5700 | 2.84 | 187.3 | 1.99 |

**Supplementary Table S1** Molecular weights and glass transition temperature (T_g_) of BioChoice lignin samples.

M_w_ refers to weight average molecular weight, M_n_ refers to number average molecular weight and PDI corresponds to polydispersity index (M_w_/M_n_). The data reported for molecular weight distribution are an average of two measurements. The reported T_g_ in this table for lignins is from the second heating scan.

DSC thermograms

**Supplementary Figure S3** DSC traces: (a-b), (c-d) and (e-f) for the first, second and third heating scan respectively: (a, c and e) soluble; and (b, d and f) insoluble fractions including the as-received lignin (ARL). Origin (Pro), version 2020. OriginLab Corporation, Northampton, MA, USA. https://www.originlab.com/

**Supplementary Figure S4** Summary of the T_gs_ as-received (ARL), soluble (ASL and ESL) and insoluble (ALR and ELR) lignin fractions representing three consecutive heating scans*.* Origin (Pro), version 2020. OriginLab Corporation, Northampton, MA, USA. https://www.originlab.com/

**Supplementary Figure S5** UV/vis spectra of (a) soluble and (b) insoluble lignin fractions including as-received lignin. The spectrum for the ARL has been reproduced in both the figures. Origin (Pro), version 2020. OriginLab Corporation, Northampton, MA, USA. https://www.originlab.com/

**Supplementary Table S2** Extinction coefficients for as-received (BioChoice lignin) and fractionated lignin samples at 280 nm.

| **Samples** | **Extinction coefficient**  **(l g^-1^cm^-1^)** |
| --- | --- |
| As-received (BioChoice) Lignin (ARL) | 27.5 |
| Acetone Soluble Lignin (ASL) | 36.1 |
| Acetone Lignin Insoluble Residue (ALR) | 25.5 |
| Ethanol Soluble Lignin (ESL) | 30.8 |
| Ethanol Lignin Insoluble Residue (ELR) | 28.5 |

**Supplementary Figure S6** ^31^P NMR of as-received lignin (ARL). TopSpin (Pro), version 3.0. Bruker, MA, USA. https://www.bruker.com/en.html

**Supplementary Table S3** Hydroxyl group content for lignin (mmol/g) calculated from the ^31^P NMR spectra.

| **Chemical**  **Shift, δ (ppm)** | **Assignment** | **OH (mmol/g)** | | | | |
| --- | --- | --- | --- | --- | --- | --- |
|  |  | **AsRL** | **ASL** | **AIL** | **ESL** | **EIL** |
| 150.0-143.3 | Total aliphatic | 1.46 | 1.08 | 1.51 | 0.64 | 0.88 |
| 145.0-140.5 | Condensed phenolic units  (C-5 substituted) | 0.54 | 1.3 | 1.14 | 1.21 | 0.13 |
| 143.0-142.0 | Syringyl (S) OH | 0.07 | 0.26 | 0.27 | 0.04 | 0.02 |
| 140.5-138.6 | Guaiacyl (G) OH | 1.23 | 1.88 | 0.59 | 0.97 | 0.51 |
| 138.5-137.6 | p-Hydroxyphenyl (H) unit | 0.09 | 0.15 | 0.14 | 0.05 | 0.02 |
| 136.4-133.6 | Carboxylic acid (COOH) | 0.52 | 0.61 | 0.37 | 0.25 | 0.13 |
| - | Total phenolic units | 1.86 | 3.33 | 1.87 | 2.23 | 0.66 |
| - | Phenolic to aliphatic ratio | 1.27 | 3.08 | 1.15 | 3.39 | 0.75 |

**Supplementary Table S4** The viscosity and electrical conductivity of specified concentrations of lignin solutions (ASL-ESL) used in the electro-spinning experiments to determine optimum concentration for electro-spinning. The solvent used in the electro-spinning experiments was a 2:1 ratio of acetone and DMSO respectively.

| **Experiment**  **number** | **ASL-ESL**  **Ratio**  **(%)** | **Total polymer concentration**  **(wt%)** | **Viscosity**  **(Pa.s)** | **Electrical conductivity**  **(Sm^-1^) x 10^-4^** | **Observation** |
| --- | --- | --- | --- | --- | --- |
| 1 | 95-5 | 48.2 (2.5 g in 3 ml) | 0.17 | 1.71 | Fused fibres |
| 2 | 95-5 | 50.4 (2.5 g in 2.75 ml) | 0.22 | 1.97 | Beaded and non-continuous fibres |
| 3 | 95-5 | 52.8 (2.5 g in 2.5 ml) | 0.42 | 2.46 | Continuous fibres |
| 4 | 95-5 | 55.4 (2.5 g in 2.25 ml) | 1.13 | 3.31 | Fused fibres |
| 5 | 90-10 | 52.8 (2.5 g in 2.5 ml) | 0.40 | 1.92 | Continuous fibres |
| 6 | 70-30 | 52.8 (2.5 g in 2.5 ml) | 0.39 | 1.89 | Continuous fibres |

The above-mentioned experiments were conducted to derive the optimum concentrations, viscosity and electrical conductivity for electro-spinning lignin.

**Supplementary Figure S7** SEM micrographs of electro-spun lignin fibres: (a-b) shows solvent rich morphology for experiment-1 (Supplementary Table **S4**) with the lowest polymer concentration; (c-d) shows morphology of beaded-fibres emanating from experiment-2; (e-f) shows smooth and circular fibre morphology and this corresponds to experiment-3 with a viscosity of 0.42 Pa.s; and (g-h) shows morphology of fused fibres from experiment-4.

**Supplementary Figure S8** SEM micrographs of electro-spun lignin fibres: (a-b) and (c-d) shows smooth and circular fibre morphology and this corresponds to experiment-5 (Supplementary Table **S4**) with a ratio of 90-10 (ASL-ESL) and experiment-6 (70-30) respectively.

1. Saito, T.; Perkins, J. H.; Vautard, F.; Meyer, H. M.; Messman, J. M.; Tolnai, B.; Naskar, A. K., Methanol Fractionation of Softwood Kraft Lignin: Impact on the Lignin Properties. *ChemSusChem* **2014,** *7* (1), 221-228.

2. Choi, J. W.; Faix, O., NMR study on residual lignins isolated from chemical pulps of beech wood by enzymatic hydrolysis. *Journal of Industrial and Engineering Chemistry* **2011,** *17* (1), 25-28.

3. Balakshin, M. Y.; Capanema, E. A., Comprehensive structural analysis of biorefinery lignins with a quantitative 13C NMR approach. *RSC Advances* **2015,** *5* (106), 87187-87199.

4. Crestini, C.; Argyropoulos, D., *Structural Analysis of Wheat Straw Lignin by Quantitative 31P and 2D NMR Spectroscopy. The Occurrence of Ester Bonds and β-O-4 Substructures*. 1997; Vol. 45.

5. Pu, Y.; Cao, S.; Ragauskas, A. J., Application of quantitative 31P NMR in biomass lignin and biofuel precursors characterization. *Energy & Environmental Science* **2011,** *4* (9), 3154-3166.

6. Sannigrahi, P.; Ragauskas, A. J.; Miller, S. J., Lignin Structural Modifications Resulting from Ethanol Organosolv Treatment of Loblolly Pine. *Energy & Fuels* **2010,** *24* (1), 683-689.

7. Rönnols, J.; Schweinebarth, H.; Jacobs, A.; Stevanic, J.; Olsson, A.-M.; Reimann, A.; Aldaeus, F., *Structural changes in softwood kraft lignin during non-oxidative thermal treatment*. 2015; Vol. 30, p 550-561.

8. Granata, A.; Argyropoulos, D. S., 2-Chloro-4,4,5,5-tetramethyl-1,3,2-dioxaphospholane, a Reagent for the Accurate Determination of the Uncondensed and Condensed Phenolic Moieties in Lignins. *Journal of Agricultural and Food Chemistry* **1995,** *43* (6), 1538-1544.
